# The NAtional randomised controlled Trial of Tonsillectomy IN Adults (NATTINA): a clinical and cost-effectiveness study: study protocol for a randomised control trial

**DOI:** 10.1186/s13063-015-0768-0

**Published:** 2015-06-06

**Authors:** Isabel Rubie, Catherine Haighton, James O’Hara, Nikki Rousseau, Nick Steen, Deborah D. Stocken, Frank Sullivan, Luke Vale, Scott Wilkes, Janet Wilson

**Affiliations:** Institute of Health and Society, Newcastle University, Newcastle upon Tyne, NE2 4AX UK; ENT Department, Freeman Hospital, Newcastle upon Tyne Hospitals NHS Foundation Trust, Newcastle upon Tyne, NE7 7DN UK; Department of Community and Family Medicine, University of Toronto, Toronto, ON M5G 1V7 Canada; Department of Pharmacy, Health and Wellbeing, University of Sunderland, Sunderland, SR1 3SD UK

**Keywords:** Tonsillitis, Sore throat, Tonsillectomy, Surgery, Randomised controlled trial

## Abstract

**Background:**

The role of tonsillectomy in the management of adult tonsillitis remains uncertain and UK regional variation in tonsillectomy rates persists. Patients, doctors and health policy makers wish to know the costs and benefits of tonsillectomy against conservative management and whether therapy can be better targeted to maximise benefits and minimise risks of surgery, hence maximising cost-effective use of resources. NATTINA incorporates the first attempt to map current NHS referral criteria against other metrics of tonsil disease severity.

**Methods/design:**

A UK multi-centre, randomised, controlled trial for adults with recurrent tonsillitis to compare the clinical and cost-effectiveness of tonsillectomy versus conservative management.

An initial feasibility study comprises qualitative interviews to investigate the practicality of the protocol, including willingness to randomise and be randomised. Approximately 20 otolaryngology staff, 10 GPs and 15 ENT patients will be recruited over 5 months in all 9 proposed main trial participating sites.

A 6-month internal pilot will then recruit 72 patients across 6 of the 9 sites. Participants will be adults with recurrent acute tonsillitis referred by a GP to secondary care. Randomisation between tonsillectomy and conservative management will be according to a blocked allocation method in a 1:1 ratio stratified by centre and baseline disease severity.

If the pilot is successful, the main trial will recruit a further 528 patients over 18 months in all 9 participating sites. All participants will be followed up for a total of 24 months, throughout which both primary and secondary outcome data will be collected. The primary outcome is the number of sore throat days experienced over the 24-month follow-up. The pilot and main trials include an embedded qualitative process evaluation.

**Discussion:**

NATTINA is designed to evaluate the relative effectiveness and efficiency of tonsillectomy versus conservative management in patients with recurrent sore throat who are eligible for surgery. Most adult tonsil disease and surgery has an impact on economically active age groups, with individual and societal costs through loss of earnings and productivity. Avoidance of unnecessary operations and prioritisation of those individuals likely to gain most from tonsillectomy would reduce costs to the NHS and society.

**Trial registration:**

ISRCTN55284102, Date of Registration: 4 August 2014

## Background

The role of tonsillectomy in the management of adult sore throat remains uncertain, and despite demonstrable compliance with prevailing empirical guidance [1], UK regional variation in tonsillectomy rates persists [2]. The 2009 Cochrane review [3] identified only 1 evaluable adult trial with just 70 participants [4] followed up for only 90 days, and the review concluded that reasonable levels of evidence to support clinical decisions were only available for children. Currently there is evidence for increasing numbers of admissions for severe or complicated tonsillitis (e.g. peritonsillar abscess) as the number of tonsillectomy operations has fallen over the past decade in England [5]. The management of sore throats costs the National Health Service (NHS) over £120 million per annum – an estimated £60 million of this for general practitioner (GP) consultations and medical therapy [6]. From 2011–12 in England alone, secondary care costs included an estimated £10 million for bed usage and around £20 million in elective adult tonsillectomy [6].

The questions that patients, doctors and health policy makers wish to answer relate to the relative costs and benefits of tonsillectomy against conservative management (delayed surgery) and whether there can be more refined surgical indications to maximise such benefits, minimise the risks and make the best use of the scarce resources that are available.

Decision-making for tonsillitis is mostly undertaken in primary care where there may be the greatest potential for evolution in the patient pathway. Tonsillectomy is a painful procedure [7], which requires an average of 14 days off work [8, 9] and has a number of less common but intrusive complications [10] including changes in taste and tongue sensation [11, 12]. Thus, irrespective of its relative merits as a treatment, like all surgical intervention it needs to be weighed carefully against the alternatives.

Antibiotic overuse in unselected community populations with viral pharyngitis is costly for health systems [13] and efforts to try to curtail antibiotic prescription in general practice are on-going [14]. However, different economic considerations apply in those selected patients with more frequent and incapacitating episodes [1]. A 3-arm comparison of immediate, versus no, versus delayed antibiotic prescribing was examined over 15 years ago in a substantial UK randomised controlled trial (RCT), which found the main effect of antibiotic use was the promotion of future medical consultations for sore throat [15]. However, the study population in that trial included substantial numbers of children [16], and the criteria for prescription were not all aligned with the Centor Clinical Prediction Rule. More importantly in the context of NATTINA, the trial related to individual index episodes of sore throat. NATTINA concerns the management of patients aged 16 years and over who have had a significant disease burden for some considerable qualifying period of time such that both they and their referring physician feel that tonsillectomy may be justified.

The NAtional Trial of Tonsillectomy IN Adults (NATTINA) consists of a feasibility study, internal pilot and definitive 9-centre randomised controlled trial with a substantial sample size of 600 adults, with embedded qualitative process evaluation. The integration of qualitative research within difficult-to-conduct randomised controlled trials is recommended to improve their feasibility, design, conduct and outcome [17]. Participants will be randomly allocated into one of two groups – surgery or conservative management. Our previous experience of a randomised trial of tonsillectomy in children [18, 19], together with other published Ear Nose and Throat (ENT) surgical trials, highlighted the problem of retaining participants in the non-surgical cohort, especially in a trial population, which was reviewed only by postal survey and diary return. These findings, along with patient and public engagement, have influenced the trial design. The concept of deferred surgery as the conservative management option rather than no surgery is more acceptable to patients. NATTINA also keeps the research team more closely engaged with the participants through two face-to-face clinic visits during follow-up and, therefore, hopes to improve compliance rates and minimise patient cross-over. The NATTINA patient involvement forum also maintains patient engagement.

There has been no known previous attempt to map the current NHS referral criteria against any other metrics of severity. NATTINA factors-in more specific and sensitive modelling of disease severity, which encourages patients to apply a simple but validated estimate of sore throat severity. Current UK surgical practice is governed by Scottish Intercollegiate Guideline Network (SIGN) guidance [1], which has hitherto been audited only to measure compliance, not validity. By carefully modelling, the costs and consequences and setting these against surrogates of baseline severity, patients, clinicians, and health service funders will be presented with a range of options as to what should be the preferred threshold for surgical intervention.

We hypothesise that NATTINA will demonstrate that more severely affected individuals, who will ultimately gain most from tonsillectomy, are more likely to be systematically and accurately characterised at an earlier stage, thus maximising the cost-effectiveness of any surgical intervention by more timely and precisely indicated intervention. Most adult tonsil disease and surgery impacts on economically active age groups, with individual and societal costs through loss of earnings and productivity. Patients will, therefore, benefit from more timely and efficient management – with less time lost from work or studies, and fewer days’ illness. The NHS will gain through lower costs with avoidance of unnecessary operations, as well as society through conservation of productivity in an economically active patient population.

## Methods/design

### Feasibility study

#### Aim

The purpose of the feasibility study is to assess the practicality of the proposed internal pilot and main NATTINA trial. The feasibility study will specifically address the key methodological issues raised via our Patient and Public Involvement (PPI) Group, as well as otolaryngology clinicians and GPs consulted to date. In-depth qualitative interviews will be used to assess the recruitment and randomisation process, design and delivery of usual care, and the experiences and acceptability of the treatments and outcome measures.

### Objectives

1. Evaluate otolaryngologists’ and recruiting specialist nurse practitioners’ willingness to randomise and randomly allocate patients to the treatment arms, as well as evaluate patients’ willingness to be randomised, taking into account the predicted variation in severity of sore throat.
2. Define clear eligibility criteria acceptable to all stakeholders.
3. Assess acceptability of conservative management treatment arm for patients whose primary care clinicians have referred them for specialist intervention.
4. Establish primary care clinicians’ willingness to refer.
5. Investigate the feasibility and acceptability of our proposed data collection methods and outcome measures. Explore illness features reported to us as of concern by our PPI Group to ensure they are captured in trial returns: e.g. chronic sore throat, night time sore throat and disruption of ability to undertake not only usual activities but also leisure pursuits. Develop participant questionnaires, weekly sore throat and Sore Throat Alert Return (STAR) data collection methods and storage.
6. Explore the processes of patient identification and recruitment. Develop patient recruitment materials including production of the standardised NATTINA randomisation DVD.
7. Establish the NATTINA patient forum.
8. Prepare three of the nine centres to be set up to start recruiting in week one of the internal pilot.

### Study setting

Patients and staff will be recruited from nine hospitals across England and Scotland.

### Target population and sample size

In-depth qualitative and cognitive interviews will be carried out by a researcher on patients, ENT staff and GPs over 5 months, or until data saturation. It is anticipated that this will require 15 ENT patients, 20 ENT staff and 10 GP interviews, but the final number is to be determined according to qualitative research design criteria. Patients will be adults with acute tonsillitis referred to otolaryngology outpatient clinics for recurrent sore throat and participating staff will be those who will be involved in the proposed NATTINA study.

### Inclusion criteria

Patients will meet the inclusion criteria for the internal pilot and main trial (see below). ENT staff will be staff members at participating sites who are likely to be involved in the proposed NATTINA trial. GPs will be practicing GPs.

### Exclusion criteria

Patients will be excluded if they meet the exclusion criteria for the internal pilot and main trial (see below). There are no exclusion criteria for ENT staff and GPs.

### Screening, recruitment and consent

#### Patients

ENT staff will identify patients attending referral visits who meet the proposed NATTINA eligibility criteria and will hand out a Participant Information Sheet (PIS) at their clinic visit. Patients who are interested in participating in an interview, or who would like more information, will complete an expression-of-interest form to hand back to the research team during the clinic visit. This form will be returned to the Newcastle Clinical Trials Unit (NCTU) team who will contact the patients and arrange an interview at a time and location convenient for them. Written informed consent will be obtained at the beginning of the interview. Feasibility study participants will not be asked to participate in the NATTINA trial.

#### ENT staff

Multiple staff (otolaryngologists, research nurses, nurse practitioners, clinic managers) who are likely to be involved in NATTINA at each of the participating sites will be given a PIS to read and will be invited for a face-to-face or telephone interview. Informed consent will be taken at the time of the interview or signed prior to interview if via telephone.

#### GPs

A sample of primary care clinicians covering catchment areas for as many participating sites as possible will be identified by the Chief Investigator (CI), primary care research networks and consultants at the nine participating sites. GPs will be contacted and provided with a PIS to read before being invited to participate in a face-to-face or telephone interview to explore their willingness to refer. Informed consent will be sought at the time of the interview or signed prior to interview if via telephone.

### Outcomes

1. Otolaryngologists’ and recruiting specialist nurse practitioners' willingness to randomise and randomly allocate patients to the treatment arms, as well as their views on the eligibility criteria, patient identification and recruitment.
2. Primary care clinicians’ willingness to refer.
3. Patients’ willingness to be randomised and their acceptability of the conservative management treatment arm, as well as views on the proposed data collection methods, including weekly sore throat alert prompts and STARs.

### Success criteria

A decision-making meeting will be scheduled for the end of the feasibility study to review the findings and to confirm that there is sufficient support from those interviewed to allow the project to continue on to the NATTINA internal pilot phase.

Success criteria for the feasibility study to allow progression to the internal pilot phase will include evidence of:

- Otolaryngology staff willing to consider participation in the study
- Potential participants also showing willingness to take part in the trial
- Eligibility criteria are acceptable and adequately defined
- Treatment pathways are acceptable and adequately defined
- Outcome measures and data collection methods are feasible and adequately defined
- Processes of patient identification and recruitment are feasible and adequately defined
- Patient information materials are refined
- NATTINA patient forum is established
- Three participating sites are able to start recruiting in week one of the internal pilot

### Data handling and record keeping

All interviews will be audio-recorded and transcribed verbatim. Anonymous audio files and transcripts will be stored electronically and kept alongside other study data.

Data will be handled, computerised and stored in accordance with the Data Protection Act 1998. Caldicott approval will be sought during set up at each participating site to enable the collection and transfer of participant information as part of this study. All study data will be stored for 5 years and in accordance with Good Clinical Practice (GCP) and NCTU Standard Operating Procedures (SOP).

### Qualitative analysis (feasibility, internal pilot and main trial)

Framework analysis will be adopted as a recommended approach for qualitative health research with objectives linked to quantitative investigation [20]. We will use NVivo software (http://www.qsrinternational.com) to aid indexing and charting. The data will be repeatedly read and coded independently by two researchers within a framework of a priori issues and those identified by participants or emerging from the data. Any divergence between coders will be discussed on an on-going basis to inform the analysis and resolve divergence in their interpretations of the data. Analysis will be discussed at regular research team meetings to identify areas for closer consideration (including negative case analysis) and to enhance credibility of the thematic framework and interpretation [21, 22]. Qualitative work will explore influences on both patient recruitment and on the implementation of the study interventions (e.g. the deferral of surgery for up to 2 years). Our analysis of barriers and facilitators to: 1) trial participation and 2) the normalisation of study interventions in clinical practice will be informed by Normalization Process Theory [23]. This model considers factors that affect implementation in four key areas; how people make sense of a new practice (coherence); the willingness of people to sign-up and commit to the new practice (cognitive participation); their ability to take on the work required of the practice (collective action); and activity undertaken to monitor and review the practice (reflexive monitoring). The approach is increasingly used in studies of the implementation of interventions in health care (www.normalizationprocess.org). In NATTINA, we will consider how well trial processes and interventions are introduced and incorporated at each site for both patient and professional (including both primary and secondary care) groups.

### Sample size calculation

Sampling will be purposive, seeking maximum variety in terms of location, age and sore throat severity among male and female participants. Sample size will be determined by reaching data saturation where the research team deem no new themes to have emerged in the 3 consecutive interviews [24]. Based on previous work by the investigators, it is estimated that this will occur at around 45+ participant interviews: 20 otolaryngology staff, 15 patients and 10 GPs.

### Internal pilot and main trial

#### Aim

The purpose of NATTINA is to establish the clinical and cost-effectiveness of tonsillectomy compared with conservative management for adult tonsillitis which, through observation and statistical modelling of outcomes, will evaluate the impact of alternative sore throat patient pathways and develop future research. The internal pilot aims to assess the ability to recruit to the main NATTINA trial and ensure robust operational processes.

### Internal pilot objectives

1. Ascertain if all trial processes, including patient identification, eligibility criteria, randomisation and data collection, work as intended and the eligibility criteria are cohesively operational.
2. Gauge more precisely the number of potential eligible patients identified in NATTINA screening clinics.
3. Investigate baseline severity spectrum through referral, recruitment and acceptability.
4. Identify barriers to patient recruitment and suggest improvements to impact on recruitment rates.
5. Measure participant compliance with the proposed weekly submission of number of sore throat days, plus STARs during sore throat episodes.
6. Identify any major emerging systematic differences between recruited participants and those who decline to participate.
7. Collate and report reasons for participation/ineligibility/decline.
8. Quantify missing data and measure attrition in sore throat data.
9. Review activity against go criterion: 6 screening clinics established, with target combined activity of 396 eligible patients screened in 6 months and target minimum n = 72 randomised.

### Main trial primary objectives

To compare the effectiveness and efficiency of tonsillectomy versus conservative management for recurrent acute tonsillitis over the 24 months following randomisation.

### Main trial secondary objectives

1. Clinical effectiveness:
  To compare other metrics of sore throat severity including responses on the Tonsillectomy Outcome Inventory 14 (TOI 14) [25] and STARs for any sore throat episodes experienced
  To compare Quality of Life (QOL) as measured by the Short Form 12 Health Survey (SF-12) [26] longitudinally during study follow-up
  To report the number of adverse events, visits to the GP/walk-in clinic/Accident and Emergency (A&E), prescriptions issued and additional interventions required for sore throats and related events through STAR data, and supported by data linkage to primary care participant records
  To adjust the estimate of effectiveness in light of other baseline covariates including severity of tonsillitis
  To evaluate the impact of alternative sore throat patient pathways by observation and statistical modelling of outcomes
  To assess to what extent trial participants are representative of the total population of sore throat patients referred to ENT clinics
2. Economic evaluation:
  To compare Quality-Adjusted Life Years (QALYs) using the Area Under the Curve (AUC) method based upon SF-6D scores derived from the SF-12 [26] responses [27] measured at baseline, throughout the study and during any episodes of sore throat experienced
  To compare the cost-effectiveness measured in terms of the incremental cost per sore throat day avoided from the perspective of the NHS and patients over 24 months
  To compare the cost-utility based on incremental cost per QALY gained from the perspective of the NHS and participants over 24 months
  To compare the cost-benefits based on the perspective of the NHS and participant’s willingness to pay to avoid a sore throat day using the NATTINA contingent valuation questionnaire ‘Value of Avoiding a Sore Throat Day’ administered at baseline and information on the number of sore throat days experienced.
3. Qualitative process evaluation: to document the views, experiences and acceptability of patients and clinicians regarding tonsillectomy and conservative management, and how patient experience may shape future research
4. Future research: to propose further research questions based on the findings of the trial analyses

### Study design and duration

This is a multi-centre, randomised, controlled surgical trial with internal pilot and embedded qualitative process evaluation, comparing immediate tonsillectomy versus conservative management (delayed surgery). The internal pilot will recruit patients over a 6-month period and, subsequent to successful completion, the main trial will commence and continue recruitment for a further 18 months.

The end of the study will be the date of the last participant’s 24-month follow-up visit and once all serious adverse events (SAEs) have been followed up.

Qualitative interviews for the qualitative process evaluation will be carried out on patients, staff and GPs during the internal pilot and at the end of the main trial.

### Intervention

Participants randomised to the treatment arm will undergo a tonsillectomy within 6–8 weeks of randomisation.

### Control

Participants in the control arm will receive conservative management and will be asked to defer surgery for up to 2 years. Conservative arm participants will receive the standard non-surgical care, as normally treated by the patients themselves or by the referring GPs in their current practice, which typically comprises self-administered analgesia plus/minus ad hoc primary care prescription of antibiotics, attendance at walk-in centres or A&E departments for more severe episodes. Participants will be reviewed at 12 months post randomisation and assessed on their willingness to remain in the delayed surgery arm.

### Study setting

We will initially set up three proposed UK research sites to start recruiting in week one of the internal pilot. This will shortly be followed by another three pilot sites. On completion of the internal pilot, a further three research sites will be set up for the main trial.

### Target population and sample size

Participants will be adults with recurrent acute tonsillitis who have been referred by their GP to secondary care.

A total of 72 participants at the 6 pilot sites will be recruited into the 6-month internal pilot. An additional 528 participants will be recruited over a further 18 months in all 9 participating sites. A total of 600 participants will be recruited throughout the study. Depending on trial progression, additional sites may be set up to aid recruitment.

In-depth qualitative interviews will be carried out with 9 ENT staff, 10 GPs and 15–20 ENT patients by a researcher as part of the qualitative process evaluation (see sample size calculation). Patients will include those that have been recruited to the NATTINA study and those who declined to participate.

### Inclusion criteria

- Age ≥ 16 years
- Recurrent sore throats that fulfil current SIGN guidance [1] for elective tonsillectomy
- Subject has provided written informed consent for participation in the study prior to any study specific procedures

### Exclusion criteria

- Under 16 years of age
- Previous tonsillectomy
- Listed directly (i.e. added to waiting list without prior elective ENT outpatient appointment) during emergency admission (e.g. due to peritonsillar abscess/quinsy)
- nability to complete self-reported questionnaires and sore throat returns
- Tonsillectomy required for other indications:
  - ■ Primary sleep breathing disorder
  - ■ Suspected malignancy
  - ■ Tonsilloliths
- Equipoise influenced materially by other clinical considerations:
  - ■ Pregnant or breastfeeding
  - ■ Bleeding diathesis
  - ■ Therapeutic anticoagulation

### Screening, recruitment and consent

The clinical team at the participating sites will identify patients who have been referred for consideration of tonsillectomy and will post a PIS along with their appointment letter. The information sheet will outline the study and how to watch the NATTINA information DVD on www.nattina.com. Screening will be performed on all patients who attend an ENT referral clinic visit with recurrent sore throat. Screening is defined as the assessment of the NATTINA eligibility criteria at the patient’s clinic visit. Potential participants who were posted a PIS will be shown the information DVD at their first clinic visit (unless already viewed online) and given the opportunity to discuss the study with the designated member of the research team. Inclusion and exclusion criteria will be checked and eligible patients invited to participate in the study.

Participants will be given reasonable time (minimum of 24 hours) to decide whether or not they would like to participate. Those who are not given a PIS before their clinic visit will receive a minimum of 24 hours to consider, and will be invited to attend a later appointment to consent. Eligible patients wishing to take part will be asked to provide written informed consent by signing and dating the Informed Consent Form (ICF), which will be witnessed, signed and dated by a member of the research team with documented, delegated responsibility to do so. The original signed consent form will be retained in the Investigator Site File, with a copy in the clinical notes and a copy provided to the participant.

Patients who, following screening, are eligible but decline to participate in NATTINA will be invited to provide anonymised baseline comparison data (age, gender, an estimate of number of sore throat days over the prior 6 months and a TOI 14 questionnaire [25]). This will allow an analysis of the comparability of the trial participants to the total pool of those referred at each of the nine sites. Declining patients will also be informed about the option to take part in an interview for the qualitative process evaluation.

### Randomisation

A blocked allocation method will be used to allocate participants to the 2 intervention groups; tonsillectomy versus conservative management, in a 1:1 ratio stratified by centre and severity. Randomisation will be administered centrally via NCTU using a secure web-based system, accessed by the Principal Investigator (PI) or delegated individual.

### Primary outcome measure

The number of sore throat days as reported through weekly ‘returns’ from the participants over a period of 24 months will be the primary outcome measure.

### Secondary outcomes measures

- Responses on the TOI 14 [25] and STAR data to measure frequency, severity, health and economic impact of any sore throat episodes experienced
- QOL as measured by the SF-12 [26] longitudinally during study follow-up
- The number of adverse events, visits to the GP/walk-in clinic/A&E, prescriptions issued and additional interventions required as collected from GP records and other primary care linkage data
- The views and experiences of patients and clinicians regarding tonsillectomy and conservative management and how patient experience may shape any future research required

### Economic outcome measures

- QALYs using the AUC method based upon SF-6D scores derived from the SF-12 [26] responses [27] measured at baseline, throughout the study and during any episodes of sore throat experienced
- Incremental cost per sore throat day avoided from the perspective of the NHS and patients over 24 months to measure the cost-effectiveness

### Data collection and follow-up

All participants will be followed up for 24 months from the date of randomisation. Once consented, the participant will complete a baseline questionnaire booklet to be returned to NCTU. The participating site will then randomise the participant via the NCTU randomisation system. Participants randomised to immediate tonsillectomy will undergo surgery within 6–8 weeks of randomisation. All NATTINA participants will be prompted weekly by their preferred method of communication (short message service (SMS) message, Email or Interactive Voice Response (IVR) via telephone) to submit the number of sore throat days experienced in the previous 7 days, and requested to submit a STAR when they experience a sore throat. Through this the participant will provide details of the severity, use of any over-the-counter and prescription medications, the nature of any professional health care advice sought and the number of hours when unable to undertake usual activities (including work/studies). An additional SF-12 [26] will also be completed relative to the episode. The research nurse will contact participants randomised to the surgical arm at 1 and 2 weeks after their tonsillectomy to collect data concerning any adverse events. The participant will be asked to attend 2 further clinic visits and complete follow-up questionnaire booklets at 12 and 24 months, and to complete 2 other questionnaire booklets at home at 6-month and 18-month interim points. The 12-month clinic visit will allow participants in the conservative management group to be assessed on their willingness to remain in the deferred group. At their final 24-month visit these participants will be asked whether they wish to go forward for tonsillectomy. Adverse events and SAEs related to the study intervention will be collected throughout the duration of the study.

Consent will be sought to access participants’ GP health records in order to gather primary health care usage data at the end of their 24-month follow-up. Data collected will cover the participant’s 24-month follow-up and 12 months prior to randomisation, and will capture adverse events, number of contacts with primary and secondary health care services, prescribing information and other relevant material to support data retrieved from STARs and post-operative research nurse telephone calls (Table 1).

**Table 1:** Data collection and time points

Measure	Baseline	Weekly (baseline to 24-month FU)	Sore throat episode	Surgery	6-month FU	12- month FU	18- month FU	24- month FU
'About You' Questionnaire	X							
'Value of Avoiding a Sore Throat Day' Questionnaire	X							
TOI 14	X				X	X	X	X
SF-12	X		X		X	X	X	X
Health Service Utilisation Questionnaire	X				X	X	X	X
Participant Time and Travel Questionnaire							X	
Comparison data (declining patients)	X							
Weekly alert		X						
Sore Throat Alert Returns (STAR)			X					
CRF	X			X	X	X	X	X
GP Linkage Data								X

*CRF* Case Report Form, *FU* follow-up, *SF-12* Short Form 12 Health Survey, *TOI 14* Tonsillectomy Outcome Inventory 14

### Qualitative process evaluation

A selection of recruited and declining patients, staff and GPs will be invited to participate in a qualitative interview to discuss their expectations, motivations for participation and their views and experiences of sore throat and of the NATTINA trial. Recruited participants who signed a consent form allowing a researcher from Newcastle University to approach them to discuss an interview will be contacted during the internal pilot or towards the end of the main trial and invited for an interview. Patients who decline to participate in the main trial will be offered an expression of interest form that they can complete and return to NCTU. A researcher will then contact the patient and arrange an interview at a time and location convenient for them. Written informed consent will be obtained at the beginning of the interview.

### Data handling and record keeping

Data will be recorded by authorised staff and stored in a secure web-based electronic data capture system (MACRO) designed and maintained by NCTU hosted on secure servers at Newcastle University. The validated database will have restricted and limited access and data transferred from site by remote access will be encrypted. Analysis of this data will be undertaken by NCTU staff. Subjects will be identified by a unique study number allocated by the randomisation system, which will be used on CRFs and questionnaire front covers. Personal details (full name, address, phone numbers, Email address and NHS number) as well as their preferred method of communication (SMS, Email, telephone) will be collected by the research nurse once the participant has been recruited, and the form faxed to NCTU via SOHO 66 (secure fax to Email system) where it will be used to send out follow-up questionnaires to participants. Selected personal information will be inserted into a database managed by an independent technology solutions company for the sole purpose of distributing weekly sore throat alert prompts and STARs centrally. Databases containing identifiable information will be secure and password protected.

All interviews for the qualitative process evaluation will be audio-recorded and transcribed verbatim. Audio files and anonymised transcripts will be securely stored in password protected files.

Data will be handled, computerised and stored in accordance with the Data Protection Act 1998. Caldicott approval will be sought during set-up at each participating site to enable the collection and transfer of participant information as part of this study. All study data will be archived for 5 years and in accordance with GCP and NCTU SOP.

### Study compliance and withdrawal

Where feasible, visit windows of ±6 weeks should ensure sufficient time is offered to facilitate scheduling appointments; non-attendance for study visits will prompt follow-up by telephone. Participants may also be contacted via telephone by the research nurse at the participating site to remind or encourage them to return questionnaires or weekly alerts. Source data verification will be performed by the Trial Manager at each participating site.

Participants will have the right to withdraw from the study at any time for any reason, and without giving a reason. The investigator will also have the right to withdraw participants from the study intervention if he/she judges this to be in their best interest. At the participants’ request, those in the conservative management arm will be able to cross over into the surgical arm or withdraw completely with no further data collected. Participants in the tonsillectomy arm will be able to either withdraw completely or cross over to the conservative management arm before surgery has occurred.

### Statistical analysis

The primary outcome measure of the total number of sore throat days reported over the 24 months of follow-up will be analysed using negative binomial regression in order to compare any change between the NATTINA arms while adjusting for the stratification variables used – recruiting centre (as a random effect) and baseline severity (as a fixed effect). This analysis will be undertaken on an intention-to-treat basis; however, participants may switch over from conservative to surgical management. In the NATTINA design, although participants are asked to commit to ‘deferred surgery’, we anticipate that a number of participants will take up the opportunity to switch to surgery. The implication of such cross over, which typifies surgical trials, is that the intention-to-treat analysis will produce a very conservative estimate of the effect of tonsillectomy. We will, therefore, undertake a planned ‘as treated’ analysis with repeated measures corresponding to two periods of follow-up for those participants who cross over from medical management to tonsillectomy. The length of these follow-up periods will act as an exposure variable in the negative binomial regression.

QOL scores based on the SF-12 will be calculated according to the scoring manual at baseline and 6, 12, 18 and 24 months post randomisation. The scores will be analysed using models developed for longitudinal data. The dependent variable will be the QOL score for an individual patient at a particular occasion. Both variation between patients and variation between occasions nested within patients will be modelled as random effects with a normal distribution. Differences between groups and changes over time will be modelled as fixed effects. The analysis will be adjusted for the differences between strata.

The analysis of other secondary outcomes will follow a broadly similar strategy; repeated measures will be analysed using a random effects model with an appropriate error structure. Should data be found to be non-normally distributed, the use of transformations or non-parametric approaches will be considered.

The true effect of tonsillectomy is likely to lie between the estimate from the intention-to-treat analysis, which is the most parsimonious account due to anticipated cross-over into surgery, and the ‘as treated’ analysis, which will tend to maximise the effect size of any surgical intervention. Outcome data analysis will be at the end of the study and for Data Monitoring and Ethics Committee (DMEC) review and will follow a full statistical analysis plan developed prior to the start of any analysis. Safety data will not be subject to statistical testing. Data with missing observations due to loss to follow-up will be examined to determine both its extent and whether it is missing at random or is informative. If data is missing to a sufficient extent, the use of appropriate multiple imputation techniques will be considered. In the event of incomplete follow-up on our primary outcome for some participants we will fit an appropriate exposure variable in the regression model.

Secondary analysis will include estimation of the effects of tonsillectomy adjusted for potentially important clinical and demographical variables.

### Economic analysis

‘Within trial’ economic analyses will be carried out from the perspective of the NHS. Costs will be based upon the costs of the randomised interventions received and on the use of subsequent care and services recorded on Case Report Forms and participant-completed questionnaires. Cost falling on participants and their families will also be recorded in a secondary analysis. Participant costs and time away from usual activities per each type of episode of care will be collected via questionnaire at 18 months. A micro costing exercise will be conducted to elicit the other resources required to estimate the costs of the surgical procedures. Other unit costs will come from nationally available data [28] and combined with use of service data to produce a cost for each trial participant. Discounting will be applied to costs and outcomes at UK recommended rates [29].

1. Cost-effectiveness analysis – based on the incremental cost per sore throat day avoided.
2. Cost-utility analysis – based on incremental cost per QALY gained. QALYs will be based upon responses to the SF-12 converted into SF-6D scores using standard algorithms. QALYs will be estimated from the responses from the SF-6D using the AUC method.
  For both the cost-effectiveness and cost-utility analyses the results will be presented as point estimates of mean incremental costs and effects as well as in stochastic analyses plots of cost and effects and cost-effectiveness acceptability curves.
3. Cost-benefit analysis – a contingent valuation method to allow participants to state their preferences in monetary values [30], to avoid a sore throat day using a participant-completed questionnaire administered at baseline. For each randomised group we will calculate mean willingness to pay and explore how valuations might vary according to participant characteristics (e.g. family income, gender, age, etc.). The data on the willingness to pay for a sore throat day avoided will be combined with information on number of sore throat days experienced and on the cost per participants. The results will be presented as point estimates and in stochastic analysis plots of cost and mean willingness to pay and incremental net benefit curves.

For all economic evaluations deterministic sensitivity analyses will be performed to explore key uncertainties. Where appropriate these analyses will be combined with a stochastic analysis with the results presented in the same ways as described above.

### Sample size calculation

By recruiting 600 participants we are allowing for a total loss to follow-up of 25 % over 24 months. Two groups of 224 participants (providing complete data at 2 years) gives 90 % power to detect an effect size of 0.33 (corresponding mean intergroup difference of 3.6 days of sore throat based on a pooled estimated standard deviation of 10.8 days) assuming a type 1 error rate of 2.5 %. The sample size calculations take account of the anticipated losses as well as predicted switch rates. We anticipate that our loss to follow-up rate should be less than the stated conservative estimate of 25 %, as we shall intensively follow-up trial participants in both arms. Underlying assumptions of the sample size calculations will be monitored by the DMEC.

Sampling for the qualitative process evaluation will be purposive, seeking maximum variety in terms of age, gender, phase of trial (pilot/main) and treatment arm (including participants who cross over). Sample size will be determined by reaching data saturation, estimated to occur at around 15–20 ENT patient interviews, 9 ENT staff interviews and 10 GP interviews.

### Study monitoring

#### Trial Management Group

The trial will be managed by a Trial Management Group (TMG) consisting of key staff members at NCTU, together with selected investigators, and will meet monthly throughout the set-up and duration of the study.

#### Trial Steering Committee

A Trial Steering Committee (TSC) has been established to provide overall supervision of the trial. The TSC consists of the CI, an independent chairperson, an independent clinician, two public members, an independent statistician and observer members of the TMG. The committee met prior to the start of the internal pilot and will convene again at the end of the internal pilot, and then annually during recruitment and for the duration of the trial.

#### Data Monitoring and Ethics Committee

An independent DMEC has been assembled to undertake independent review and will monitor efficacy and safety endpoints. The committee consists of an independent chairperson, an independent clinician and an independent statistician and has met for the first time to discuss and advise on the inclusion of an interim analysis and possible adoption of a formal stopping rule for efficacy or safety. The committee will meet at the end of the internal pilot and at least annually throughout the course of the trial.

#### Patient and Public Involvement Group

The PPI Group will consist of several ENT patients and will meet annually with a researcher from Newcastle University. The group will act as a research advisory group to discuss the design of NATTINA and any issues that have occurred. PPI members will also be contacted via Email for more urgent matters.

#### Ethical approval and confidentiality

The conduct of this study will be in accordance with the recommendations for physicians involved in research on human subjects adopted by the 18th World Medical Assembly, Helsinki 1964 and later revisions. Favourable ethical opinion from an appropriate Research Ethics Committee (REC) (North East – Tyne & Wear South) has been obtained. NHS Research and Development (R&D) approvals will be obtained at each site before recruitment can commence. R&D approval will be obtained from the following: The Freeman Hospital, Newcastle, Sunderland Royal Hospital, Ninewells Hospital and Tayside Children’s Hospital, Dundee, Glasgow Royal Infirmary, Aberdeen Royal Infirmary, Bradford Royal Infirmary, Queen Elizabeth Hospital, Birmingham, and Guy’s Hospital, London before recruitment can commence. NCTU will require a written copy of local approval documentation before initiating each centre and accepting participants into the study. PISs will be provided to all referred patients and written informed consent will be obtained prior to randomisation and any study interventions.

Personal data will be regarded as strictly confidential. To preserve anonymity, a unique participant ID will be assigned to each participant at randomisation. Participants will be made aware via the PIS, and will give consent for their contact details (name, address, phone numbers and Email address) to be used by NCTU to send out questionnaires, and by our commercial partner to send out weekly alert prompts and STARs. Participants will sign a consent form giving their permission for a researcher from Newcastle University to invite them for an optional interview. Declining patients will only be contacted by the researcher if an expression of interest form has been returned. ENT staff who are invited for interview will already be involved in the study at the participating sites. GP contacts will be identified by the CI and PI from each participating site, and invited for interview.

Written consent will be sought from participants to allow access to their electronic GP records for primary health care linkage data. The participant’s NHS number, along with their initials and date of birth, will be used to link primary care data to the participant’s ID. No personal identifiable information (other than initials and date of birth) will be transferred from the GP records onto the NATTINA database.

All electronic personal information will be kept on password protected databases with restricted access and paper forms securely filed in a locked cabinet. Only the clinical team at the participating sites will have access to key data, which links study identifiers to individual datasets. The study will comply with the Data Protection Act, 1998. All study records and Investigator Site Files will be kept at site in a locked filing cabinet with restricted access.

## Discussion

The role of tonsillectomy for recurrent sore throat in adults is still uncertain, and current statistics demonstrate a decrease in the number of tonsillectomies in recent years with an increase in the number of emergencies [5, 6]. Both the NHS and primary health care services require more evidence-based referral guidelines for patients with recurrent acute tonsillitis. NATTINA will estimate treatment effectiveness based on number of sore throat episodes, sore throat severity, QOL and number of primary/secondary health care interactions at baseline and throughout the participant’s 24-month follow-up. The study will also consider socio-economic aspects and estimate the relative efficiency to the NHS and patients. Incorporated qualitative interviews will collect valuable views and experiences from patients, staff and referring clinicians to shape guidelines and future decision-making for tonsillitis patients. The amalgamation of NATTINA outcome measures will facilitate the modelling of future patient sore throat pathways and provide accurate guidelines for tonsillectomy referral.

## Trial status

The feasibility study has now been completed and the first recruiting clinic for the NATTINA trial is planned to start in April 2015.

## Abbreviations

A&E: Accident and Emergency
AUC: Area Under the Curve
CI: Chief Investigator
CRF: Case Report Form
DMEC: Data Monitoring and Ethics Committee
ENT: Ear Nose and Throat
GCP: Good Clinical Practice
GP: general practitioner
HTA: Health Technology Assessment
ICF: Informed Consent Form
IVR: Interactive Voice Response
NCTU: Newcastle Clinical Trials Unit
NHS: National Health Service
NIHR: National Institute for Health Research
PI: Principal Investigator
PIS: Participant Information Sheet
PPI: Patient and Public Involvement
QALY: Quality-Adjusted Life Year
QOL: Quality of Life
R&D: Research and Development
RCT: randomised controlled trial
REC: Research Ethics Committee
SAE: serious adverse event
SF-12: Short Form 12 Health Survey
SF-6D: Short Form 6 Dimensions
SIGN: Scottish Intercollegiate Guideline Network
SMS: short message service
SOP: Standard Operating Procedures
STAR: Sore Throat Alert Return
TMG: Trial Management Group
TOI 14: Tonsillectomy Outcome Inventory 14
TSC: Trial Steering Committee
